# A case report of neurosarcoidosis mimicking Guillain-Barré syndrome: the diagnostic utility of skin biopsy in neurosarcoidosis

**DOI:** 10.1186/s12883-026-04753-4

**Published:** 2026-02-19

**Authors:** Kaede Ishikawa, Kazuhiro Horiuchi, Sho Saika, Shuntaro Nakamura, Sumire Nunomura, Kazuki Yamada, Masaaki Yoshikawa, Haruki Koike, Ichiro Yabe

**Affiliations:** 1 Department of Neurology, Hakodate Municipal Hospital, 1-10-1, Minato-cho, Hakodate, Japan; 2 Department of Neurology, Japanese Red Cross Asahikawa Hospital, Asahikawa, Japan; 3 Department of Neurology, Faculty of Medicine, Graduate School of Medicine, Hokkaido University, Sapporo, Japan; 4 Department of Neurology, Saga University, Saga, Japan

**Keywords:** Sarcoidosis, Neurosarcoidosis, Guillain-Barré syndrome, Skin biopsy, Peripheral neuropathy

## Abstract

**Background:**

We report a compelling case of neurosarcoidosis in a 74-year-old Japanese woman. She initially presented with a clinical course that closely mimicked Guillain-Barré syndrome (GBS), and the definitive diagnosis of neurosarcoidosis was eventually made through a skin biopsy.

**Case presentation:**

The patient presented with progressive limb weakness and sensory disturbances and was initially treated for a presumptive diagnosis of GBS. Although her condition showed an initial temporary improvement, her symptoms subsequently relapsed and worsened. This atypical and relapsing clinical pattern prompted a comprehensive systemic workup to find an alternative diagnosis. Based on the histological findings on skin biopsy and the clinical presentation, a diagnosis of probable neurosarcoidosis was made.

**Conclusion:**

This case highlights the importance of considering neurosarcoidosis in the differential diagnosis of GBS with an atypical course. A comprehensive systemic workup is crucial for such cases. It further emphasizes that a thorough systemic evaluation is essential for accurate diagnosis, as a simple and accessible procedure like a skin biopsy can provide essential histological evidence to support the diagnosis.

## Background

Sarcoidosis is a multisystem inflammatory disease characterised by the formation of non-caseating granulomas, affecting various organs such as the lungs, heart, eyes, skin, and nerves.

Neurosarcoidosis occurs in approximately 5–10% of patients with sarcoidosis and presents with diverse clinical manifestations, including cranial neuropathies, meningeal involvement, central nervous system lesions, myopathy, and peripheral neuropathy [1].

Peripheral nerve sarcoidosis is reported in 15% of cases of neurosarcoidosis [2]. Neurosarcoidosis is often difficult to definitively diagnose due to the challenges of obtaining a biopsy. In some cases, non-caseating granulomas cannot be confirmed through peripheral nerve biopsy [3]. Neurosarcoidosis can present as acute or subacute polyradiculitis, producing a clinical picture similar to that of Guillain–Barré syndrome (GBS). A pre-existing diagnosis of sarcoidosis is present in only a minority of patients at the onset of peripheral neuropathy; more commonly, the neuropathy serves as the presenting clinical feature of the disease [4]. In neurosarcoidosis, pathological biopsy is more difficult to obtain than in sarcoidosis of other organs, which makes the initial diagnosis even more challenging.

We report a case initially treated as GBS that experienced multiple recurrences and was ultimately diagnosed with probable neurosarcoidosis, supported by histological evidence of systemic sarcoidosis from a skin biopsy. This case highlights the need to consider neurosarcoidosis in the differential diagnosis of patients with GBS-like symptoms showing an atypical course, and the potential diagnostic utility of skin biopsy in establishing a systemic diagnosis.

## Case presentation

We report a case of peripheral nerve sarcoidosis in a 74-year-old Japanese woman. She had a history of hypertension and chronic lower back pain and was taking antihypertensive and analgesic medications. She had no family history of neuromuscular disease and no known allergies to food or drugs.

Ten days prior to admission (Day 0), she developed weakness in both lower limbs, numbness in both upper limbs, and sensory loss in her right foot. There were no precipitating factors, such as a preceding respiratory or gastrointestinal infection or recent vaccination. The patient denied acute neck pain, worsening of her chronic back pain, bowel or bladder dysfunction, or autonomic symptoms. Constitutional symptoms, including fever and weight loss, were also absent. The weakness gradually worsened, making it difficult for her to stand, leading to her referral to our hospital on Day 10.

On examination, her vital signs were within normal limits. Lung sounds were clear, the abdomen was flat with no tenderness, and no skin rash was observed.

Neurological examination revealed generalized muscle weakness affecting both proximal and distal muscles of all four limbs, graded 3/5 on the Medical Research Council (MRC) scale. Muscle bulk and tone were preserved in all extremities. Deep tendon reflexes showed generalized hyporeflexia. Sensory examination demonstrated decreased tactile and pain sensation from the fingertips to the wrists bilaterally, with right-sided predominance, as well as decreased pain sensation from the toes to the ankles. Position sense was also found to be impaired in the distal joints of all four limbs. Romberg’s test could not be performed because the patient was unable to maintain an independent standing position.

Initial laboratory investigations, including full blood count, renal function, and C-reactive protein, were within normal limits. The creatine kinase (CK) level was normal at 88 IU/L (reference range: 32–180 IU/L). Although mild elevations in aspartate aminotransferase 117 U/L (reference range: 7–38 U/L) and alanine aminotransferase 84 U/L (reference range: 4–43 U/L) were noted, blood glucose and HbA1c levels were within normal limits. Given the clinical presentation of acute-onset neuropathy with non–length-dependent sensorimotor involvement, antinuclear antibody (ANA) testing was performed to evaluate for autoimmune etiologies. The ANA titer was elevated at 1:640 (normal: <1:40), while other serological markers, including anti-SS-A, PR3-ANCA, MPO-ANCA, and M-protein, were negative. Serum IgM anti-GM1 antibodies were measured because Guillain–Barré syndrome was initially suspected and were found to be weakly positive.

CSF analysis revealed a lumbar opening pressure of 115 mmH_2_O, a cell count of 12 cells/µL (with 11 lymphocytes and 0 neutrophils), a protein level of 52.1 mg/dL (reference range: 10–40 mg/dL), a glucose level of 53 mg/dL, an immunoglobulin G (IgG) level of 7.9 mg/dL, and an IgG index of 0.61 (reference range < 0.73). Spinal fluid cultures were negative for both bacteria and fungi.

**Fig. 1:**
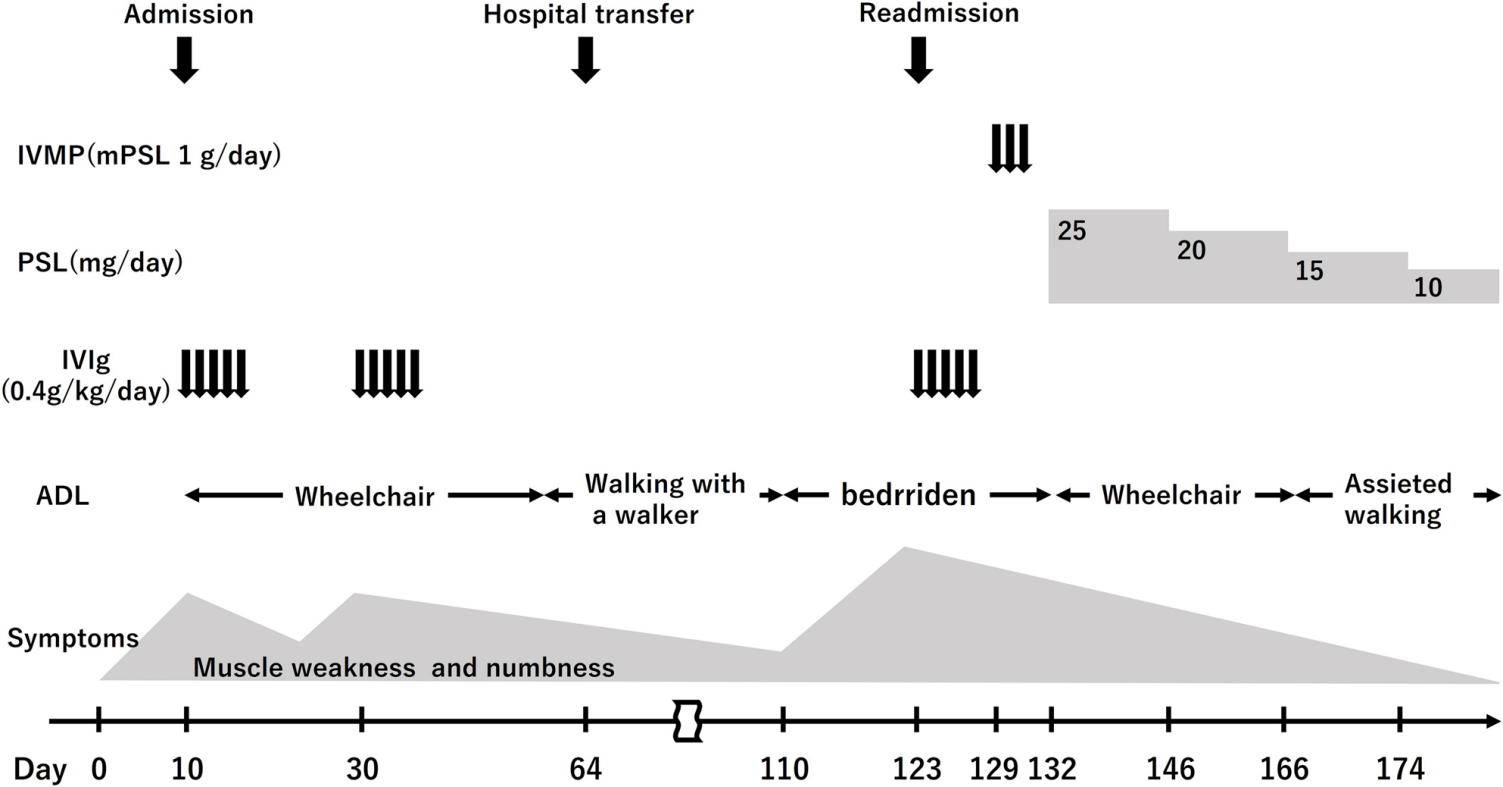
Clinical course of the patient. A patient initially suspected of having Guillain-Barré syndrome received two courses of IVIg. The patient’s symptoms initially improved but later recurred, causing the patient to become bedridden. A subsequent skin biopsy established a diagnosis of peripheral nerve sarcoidosis. Following the administration of IVIg and corticosteroids, the patient’s ambulation was restored. IVMP, intravenous methylprednisolone ; PSL, prednisolone ; IVIg, intravenous immunoglobulin

To screen for occult malignancy and systemic sarcoidosis, computed tomography (CT) of the chest, abdomen, and pelvis was performed. The scan revealed small lymph nodes in the mediastinum, but no malignant tumors were identified. MRI scans of the brain and spinal cord were normal. No enlargement of the nerve roots was observed. On day 11, nerve conduction studies (NCS) demonstrated reduced amplitudes of the compound muscle action potential (CMAP) in both ulnar nerves (right: 1.3 mV, left: 1.1mV, reference range: ≧2.8 mV) and both peroneal nerves (right: 790 µV, left: 310 µV, reference range: ≧2.5 mV). Nerve conduction velocities were within normal limits. Sensory nerve action potential (SNAP) of both ulnar nerves and both sural nerves were not detectable. Needle electromyography and H-reflex studies were not performed. The clinical course is shown in Fig. 1.

Initially, an axonal variant of Guillain–Barré syndrome (GBS), such as acute motor–sensory axonal neuropathy (AMSAN), was considered based on the acute onset of symmetrical limb weakness, reduced deep tendon reflexes, and nerve conduction study findings showing decreased compound muscle action potentials (CMAPs). However, the presence of cerebrospinal fluid pleocytosis was recognized as an atypical feature for classical GBS. Given the initial clinical suspicion, the patient was treated with intravenous immunoglobulin (IVIg) at a dose of 0.4 g/kg/day for five days, from Day 10 to Day 14.

Immediately after treatment, there was an improvement in limb muscle weakness (MRC score 4/5), and the patient was able to walk with assistance. Numbness in both lower limbs improved as well. However, despite ongoing rehabilitation, limb muscle weakness worsened to 3/5 on the Medical Research Council (MRC) scale, and numbness increased on Day 24. This deterioration, occurring 14 days after completion of the first course of intravenous immunoglobulin (IVIg), was initially interpreted as a treatment-related fluctuation (TRF), although TRF is considered atypical in the clinical course of GBS/AMSAN. Given this interpretation, the patient subsequently received a second five-day course of IVIg from Day 30 to Day 34.

After the second immunoglobulin treatment, muscle weakness and numbness in the limbs improved. The patient was transferred to another hospital for rehabilitation on Day 64. The patient initially regained the ability to walk with a walker; however, muscle weakness and numbness in the limbs worsened on Day 110. The patient was subsequently readmitted to our hospital on Day 123.

On neurological examination at the time of transfer, the patient presented with left ptosis, which was clinically interpreted as a partial left oculomotor (III) nerve palsy. Muscle weakness was present in both the proximal and distal muscles of all extremities, graded 3/5 on the MRC scale, and deep tendon reflexes were absent in all extremities. Sensory examination revealed diminished pain sensation extending from the shoulders to the distal upper limbs and from the inguinal regions to the distal lower limbs bilaterally. Severe impairment of position sense was noted in the distal joints of all four limbs. Due to severe sensory ataxia exacerbated by the motor weakness, the patient was unable to maintain a sitting or standing position and was bedridden.

Blood tests showed a serum angiotensin-converting enzyme(ACE) level of 22.4 U/L (reference range: 8.3–21.4 U/L) and soluble interleukin-2 receptor (sIL-2R) level of 1,471 U/mL (reference range: 204–587 U/mL). The sIL-2R level was measured as a marker of systemic disease activity of sarcoidosis. Serum calcium levels were within the reference range, while a 24-hour urine calcium collection was not performed. Paraneoplastic syndrome–related antibodies were assessed to exclude paraneoplastic neurological syndromes and were negative.

CSF analysis revealed a lumbar opening pressure of 115 mmH_2_O, a cell count of 4 cells/µL (lymphocytes 3, neutrophils 1), a protein level of 34.7 mg/dL, a glucose level of 47 mg/dL, an IgG level of 6.7 mg/dL, and an IgG index of 0.47 (reference range < 0.73). CSF ACE was not measured. CT showed no evidence of malignancy, but there was hilar lymphadenopathy. Contrast-enhanced brain MRI was normal. Contrast-enhanced MRI of the trunk showed no enhancement of the cauda equina or nerve roots. On day 125, nerve conduction studies revealed decreased CMAP amplitudes in both median, ulnar, tibial, and peroneal nerves. Nerve conduction velocities were mildly reduced. The SNAPs of both ulnar nerves were reduced (right: 0.8 µV, left: 6.4 µV, reference range: ≧18 µV), with corresponding reductions in nerve conduction velocity (right: 33.3 m/s, left: 26.4 m/s, reference range: ≧44 m/s). SNAPs of both median and both peroneal nerves were not recordable. F-waves were poorly elicited, but no evidence of demyelination, such as prolonged distal latency or conduction block, was observed. Needle electromyography and H-reflex studies were not performed.

**Fig. 2:**
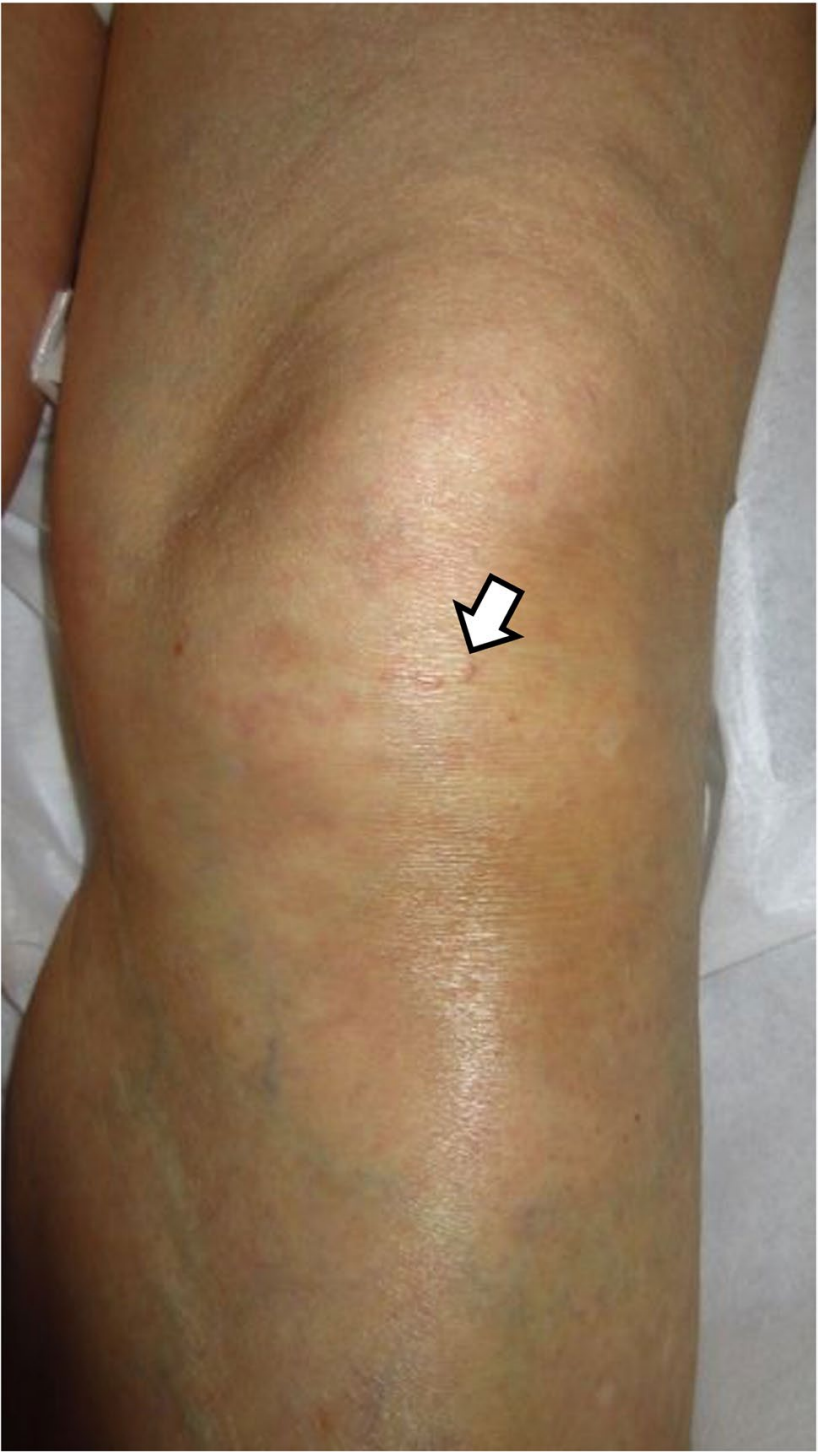
Skin findings. A red papule with scar infiltration was observed on the extensor surface of the left knee. The arrow indicates the papule

**Fig. 3:**
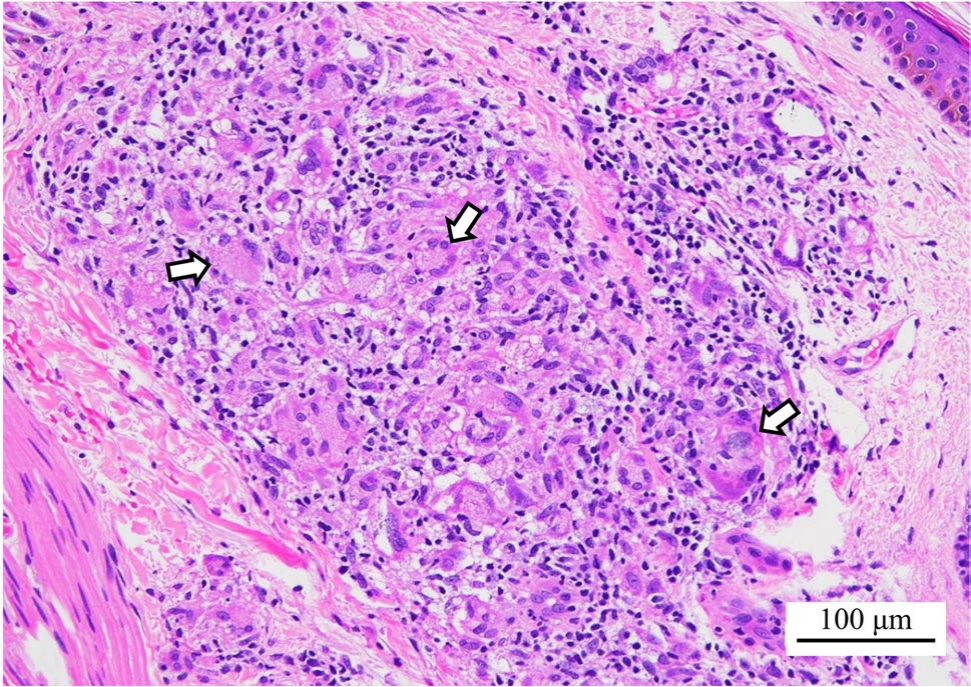
Pathological findings of the skin biopsy. Hematoxylin and eosin (H&E) staining revealed non-caseating granulomas with multinucleated giant cells in the dermis. Arrows indicate multinucleated giant cells

Cardiac echocardiogram, upper endoscopy, and colonoscopy were all unremarkable. No uveitis was observed. Although no skin rash was observed at the initial visit, a red papule with scar infiltration was noted below the left knee (Fig. 2), and a biopsy revealed non-caseating granulomas containing multinucleated giant cells (Fig. 3). Although mild hilar lymphadenopathy was present, bronchoalveolar lavage and transbronchial lung biopsy were not performed because there were no respiratory symptoms or parenchymal lung involvement, and the patient was bedridden.

**Fig. 4:**
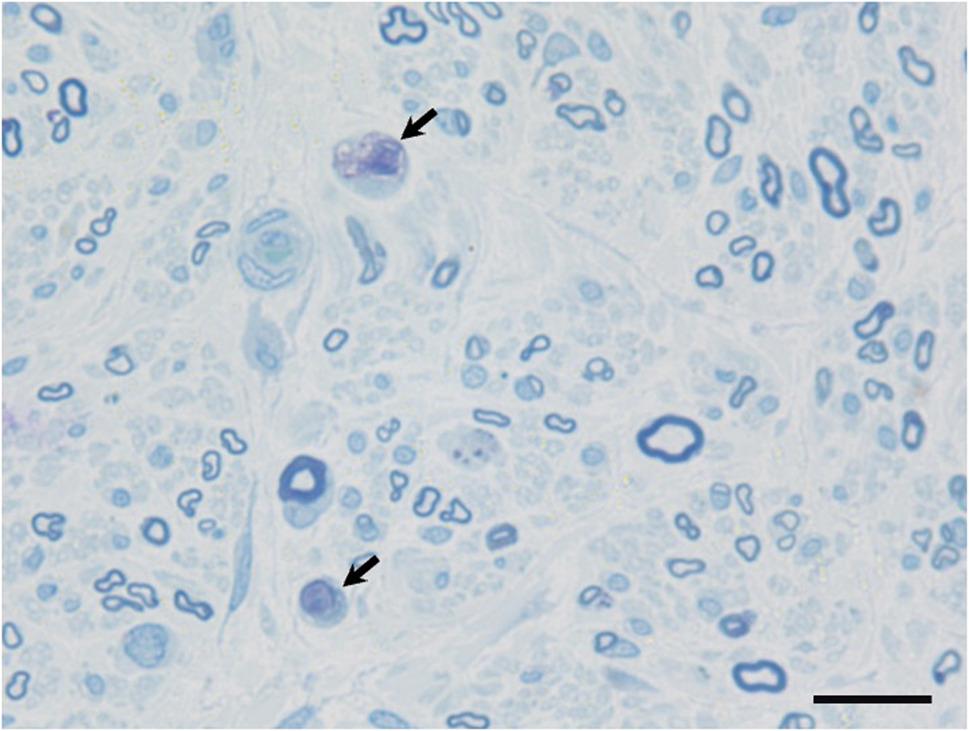
Pathological findings of the sural nerve. A sural nerve biopsy was performed on the right side. Toluidine blue staining sections revealed a predominant loss of large myelinated fibers. Arrows indicate myelin ovoids suggestive of axonal degeneration. No evidence of demyelination, such as thinly myelinated fibers or onion bulbs, was found

Right sural nerve biopsy demonstrated axonal degeneration predominantly involving large fibers, without granulomas (Fig. 4).

Gallium scintigraphy was performed following treatment and demonstrated no evidence of abnormal uptake. ¹⁸F-FDG PET imaging was not performed. Based on these findings, a diagnosis of probable neurosarcoidosis was made.

After transfer, treatment consisted of intravenous immunoglobulin (IVIg; 0.4 g/kg/day for 5 days) from Day 123, and intravenous methylprednisolone (1 g/day for 3 days) from Day 129. Following the treatment, the patient was able to sit up unaided, and both muscle strength and numbness in the limbs improved. Prednisolone was then administered at 25 mg/day (0.5 mg/kg), along with weekly methotrexate at 4 mg. The patient experienced no relapse thereafter and was able to ambulate with minimal assistance at discharge on Day 166.

The patient’s functional status, assessed using the modified Rankin Scale (mRS), was 4 on admission and improved to 2 following treatment.

## Discussion and Conclusions

We reported a rare and instructive case of a patient initially treated for GBS who experienced repeated relapses, eventually leading to a diagnosis of probable neurosarcoidosis, supported by a skin biopsy. The patient’s subacute progression made differentiation from GBS, particularly its subtypes AMAN and AMSAN, exceptionally challenging in the early stages of the disease.

Peripheral nerve sarcoidosis is a rare disease that often presents with subacute progressive radiculopathy, a clinical course that can mimic GBS. Although it frequently leads to axonal damage, demyelinating changes and conduction block can also be observed [5, 6]. Furthermore, elevated ACE levels in serum and CSF are present in only a minority of cases [7]; in the present case, while the serum ACE level was mildly elevated, CSF ACE was not measured. The presence of anti-ganglioside antibodies, which are characteristic of GBS, also provided a basis for the initial GBS suspicion. Similarly, cases of peripheral nerve sarcoidosis presenting with a GBS-like course and axonal damage have been reported to be positive for anti-GalNAc-GD1a antibodies [8]. The IgM-GM1 antibody was weakly positive in this case, which was considered an incidental finding.

In this case, key findings for distinguishing the condition from GBS included a clinical course of multiple relapses spanning more than four weeks and a mild pleocytosis in the CSF. It is well-established that the clinical progression of GBS typically halts within four weeks of symptom onset [9]. Therefore, in cases where progression continues beyond four weeks or when relapses are observed, the possibility of other peripheral neuropathies should be considered. Furthermore, CSF analysis in GBS characteristically reveals albuminocytologic dissociation, with a lack of significant cellular increase. In contrast, pleocytosis in the CSF is a frequently reported finding in peripheral nerve sarcoidosis [10]. Accordingly, even in patients presenting with a GBS-like course, the presence of an elevated CSF cell count should prompt consideration for further diagnostic investigation.

The difficulty in differentiating peripheral nerve sarcoidosis from GBS often stems from the challenge of obtaining a definitive pathological tissue sample. Reports indicate that biopsy of a nerve lesion is difficult in 50% of neurosarcoidosis cases, with the diagnosis being made from lesions in other organs [11]. Furthermore, as seen in our case, even when a nerve biopsy is performed, there are reported instances of peripheral nerve sarcoidosis where non-caseating granulomas cannot be demonstrated [3].

The most common extracranial organ involvement in neurosarcoidosis is mediastinal lymphadenopathy [12]. Our patient exhibited enlarged mediastinal lymph nodes; however, after consultation with the pulmonology department, bronchoalveolar lavage and transbronchial biopsy were not performed. This decision was based on the patient’s bedridden condition and the fact that a diagnosis had already been histologically established by skin biopsy, rendering additional invasive procedures disproportionately risky. Cutaneous lesions are observed in approximately 25–30% of patients with sarcoidosis, including scar infiltration, as seen in our patient [13]. In such cases, skin biopsy represents a simple and minimally invasive diagnostic approach that can be performed safely in many clinical settings.

While GBS was initially considered the most likely diagnosis based on the clinical presentation, its atypical course—including multiple relapses and increased cerebrospinal fluid cell counts—prompted further investigation. This ultimately led to a diagnosis of probable neurosarcoidosis supported by the histological findings from a skin biopsy. This case highlights the importance of considering alternative diagnoses like neurosarcoidosis in patients with GBS-like symptoms, especially when atypical features are observed. A thorough, systematic evaluation extending beyond neurological symptoms to include other systems, such as the lungs, skin, and eyes, is crucial. Although a skin biopsy does not strictly prove the presence of granulomas within the nerve tissue, it serves as a pivotal and minimally invasive tool for identifying systemic sarcoidosis in patients with compatible neurological manifestations.

The treatment of neurosarcoidosis primarily involves corticosteroids as first-line therapy. Oral prednisolone (PSL) at a dose of 0.5–1.0 mg/kg/day is typically administered, while high-dose intravenous corticosteroid pulse therapy is considered for severe cases, followed by a transition to oral PSL with gradual tapering. In patients with relapsing or steroid-dependent disease, second-line immunosuppressive agents, such as methotrexate, azathioprine, or mycophenolate mofetil, are commonly used [14]. In refractory cases, tumor necrosis factor-alpha (TNF-α) inhibitors, particularly infliximab, have been established as highly effective third-line therapies. Recent studies have demonstrated that infliximab is associated with high clinical response rates and enables corticosteroid dose reduction in patients with severe or progressive neurosarcoidosis [15]. In the present case, although initial treatment with intravenous immunoglobulin (IVIg) provided only transient improvement, a clear therapeutic response was achieved with corticosteroids and methotrexate. This clinical course is consistent with the expected treatment responsiveness of neurosarcoidosis.

We reported a case of peripheral neuropathy due to probable neurosarcoidosis in which skin biopsy played a key role in establishing the diagnosis. The patient was initially suspected of having GBS but showed an atypical relapsing course. This case suggests that neurosarcoidosis should be considered as a crucial differential diagnosis in GBS patients who present with atypical features such as multiple relapses or pleocytosis. Careful skin examination followed by biopsy represents a useful and minimally invasive approach to support the diagnosis of neurosarcoidosis.

## Data Availability

All data generated or analyzed during this study are included in this published article.

## Abbreviations

ACE: Angiotensin-Converting Enzyme
AMAN: Acute Motor Axonal Neuropathy
AMSAN: Acute Motor-Sensory Axonal Neuropathy
ANA: Antinuclear Antibody
BAL: Bronchoalveolar Lavage
CMAP: Compound Muscle Action Potential
CSF: Cerebrospinal Fluid
CT: Computed Tomography
GBS: Guillain-Barré Syndrome
IVIg: Intravenous Immunoglobulin
IVMP: Intravenous Methylprednisolone
MPO-ANCA: Myeloperoxidase-Antineutrophil Cytoplasmic Antibody
MRC: Medical Research Council
MRI: Magnetic Resonance Imaging
mRS: Modified Rankin Scale
NCS: Nerve Conduction Studies
PR3-ANCA: Proteinase-3-Antineutrophil Cytoplasmic Antibody
PSL: Prednisolone
sIL-2R: Soluble Interleukin-2 Receptor
SNAP: Sensory Nerve Action Potential
SS-A: Sjögren’s Syndrome-A
TRF: Treatment-Related Fluctuation
